# Immuno-Oncology biomarkers 2010 and beyond: Perspectives from the iSBTc/SITC biomarker task force

**DOI:** 10.1186/1479-5876-8-130

**Published:** 2010-12-07

**Authors:** Lisa H Butterfield, Mary L Disis, Samir N Khleif, James M Balwit, Francesco M Marincola

**Affiliations:** 1 Departments of Medicine, Surgery and Immunology, University of Pittsburgh, Pittsburgh, PA, USA; 2 Tumor Vaccine Group, Division of Oncology, University of Washington, Seattle, WA, USA; 3 Cancer Vaccine Section, National Cancer Institute, National Institutes of Health, Bethesda, MD, USA; 4 Society for Immunotherapy of Cancer and Executive Director, Inc., Milwaukee, WI, USA; 5 Infectious Disease and Immunogenetics Section (IDIS), Dept. of Translation Medicine, Clinical Center, and Center for Human Immunology (CHI), National Institutes of Health, Bethesda, MD, USA

## Abstract

The International Society for Biological Therapy of Cancer (iSBTc, recently renamed the Society for Immunotherapy of Cancer, SITC) hosted a one-day symposium at the National Institutes of Health on September 30, 2010 to address development and application of biomarkers in cancer immunotherapy. The symposium, titled *Immuno-Oncology Biomarkers 2010 and Beyond: Perspectives from the iSBTc/SITC Biomarker Task Force*, gathered approximately 230 investigators equally from academia, industry and governmental/regulatory agencies from around the globe for panel discussions and presentations on the following topics: 1) immunologic monitoring: standardization and validation of assays; 2) correlation of immunity to biologic activity, clinical response and potency assays; 3) novel methodologies for assessing the immune landscape: clinical utility of novel technologies; and 4) recommendations on incorporation of biomarkers into the clinical arena. The presentations are summarized in this report; additional program information and slides are available online at the iSBTc/SITC website.

## Introduction

Over the last decade, cancer therapies that target specific molecular pathways or specific cell types have moved from the laboratory into clinical practice. Similarly, biomarkers that may indicate suitable patient populations for these therapies or act as surrogates for the potential development of a clinical response are increasingly used in the clinic. The clinical application of biomarkers to assess the effect of immune-based cancer therapies is important for several reasons. First, immune-based treatments, such as vaccines, are often designed to elicit a specific response so that the measurement of that response could be a marker of product (e.g., vaccine) potency. Secondly, as immune-based therapies are tested earlier in the therapeutic pathway (e.g., in the adjuvant setting), biomarkers of response become increasingly important as potential endpoints of clinical trials. Finally, clinically qualified biomarkers are needed so that new immunotherapies can be rapidly and efficiently tested and translated to clinical practice.

As laboratory-based assays are being transitioned to clinical assays, several issues are raised. The assays must be robust. The clinical samples collected for analysis must be processed in a uniform way to ensure reproducibility of results. Results must be reported in a detailed and uniform way. New assays which have been developed, that will allow broad analysis of multiple immune parameters, must now be better utilized. The lessons learned from biomarker studies in fields such as HIV/AIDS and other infectious diseases, must be better incorporated into cancer immunotherapy studies.

To address these and other issues related to the development and application of biomarkers in cancer immunotherapy, the International Society for Biological Therapy of Cancer (iSBTc, recently renamed the Society for Immunotherapy of Cancer, SITC) hosted a one-day symposium at the National Institutes of Health on September 30, 2010. The symposium, titled *Immuno-Oncology Biomarkers 2010 and Beyond: Perspectives from the iSBTc/SITC Biomarker Task Force*, was organized by Lisa H. Butterfield, PhD (University of Pittsburgh), Mary L. Disis, MD (University of Washington), Samir N. Khleif, MD (National Cancer Institute, CCR) and Francesco Marincola, MD (National Institutes of Health, CC, DTM). This program was a direct extension of the efforts of the iSBTc/SITC Biomarkers Taskforce [1,2], which recently published a collaborative report of its 2009 Workshop (*iSBTc-FDA-NCI Workshop on Prognostic and Predictive Immunologic Biomarkers in Cancer*) and the recommendations which resulted from the work of the Taskforce [3].

SITC President Bernard A. Fox, PhD (Earle A. Chiles Research Institute) initiated the symposium with a presentation on critical hurdles in cancer immunotherapy that lead to delays of scientific discoveries which provide strong evidence of antitumor effects in preclinical models to be tested in patients. As an extension from the 2009 iSBTc-FDA-NCI Workshop on Biomarkers, SITC and collaborating organizations had identified seven critical hurdles to the effective translation of cancer immunotherapy: 1) the inadequacy of animal models as predictors of efficacy; 2) the prolonged time to obtain approval for clinical trials; 3) the complexity of cancer biology/immunology; 4) the inability to obtain approval to combine most promising new agents in trials; 5) the lack of definitive biomarker(s) for assessment of clinical efficacy; 6) the paucity of translational research teams; and 7) the insufficient exchange of information critical to advancing the field. Fox discussed each of these problems and stressed the need to intensify collaboration to define potential solution. Accordingly, following the symposium (October 1, 2010) SITC hosted a Collaboration Summit with representatives from nine other domestic and international associations with similar interests in promoting research and translation of cancer immunotherapy (see Appendix). In an effort spearheaded by Fox, on behalf of SITC, the collaborating associations are preparing a joint publication that further defines these critical hurdles to cancer immunotherapy and joint initiatives to overcome the identified barriers.

Samir N. Khleif, MD (National Cancer Institute, Center for Cancer Research) spoke briefly on the priorities in biomarker development in immunotherapy. He started by identifying the gaps between the ideal setting/goals of immunotherapy, its current state, and the role that biomarkers may play to bridge such gaps. He outlined the current state of immunotherapy/vaccine approaches as highly empirical in their design, which is partly a result of the lack of full understanding of the immune system response to therapy and its consequent interaction with the tumor microenvironment; and the lack of understanding of effective immune endpoints measurements. He described the complexity of immunotherapeutics compared to other types of cancer-targeted therapy for the need of immunotherapy agents to interact with the immune system, tumor microenvironment, and the tumor, to be able to generate a meaningful clinical response. This further reflects the complexity of developing biomarkers for immunotherapy and the need for a wider array of biomarkers that goes beyond the standard needed for development of cancer-targeted therapy (diagnostic, predictive, metabolism and outcome biomarkers). Immunotherapy may also require selecting biomarkers (e.g., to identify patients expressing a specific antigen and the ability to express the antigen), and biologic response biomarkers that determine the ability to generate an immune response to the therapy, which is needed for tumor response. He also addressed the complex variability of the "effective" immune response biomarkers and what biomarkers would predict the susceptibility for the generation of an effective immune response.

A major effort is required to integrate immune profile biomarkers within the clinical trial design with better strategies to correlate objective responses. Further, a biomarker development process should be defined. Khleif concluded his presentation with the identification of the following critical areas for biomarker development: biospecimens; analytical performance/validation; standardization and harmonization; collaboration and data sharing; regulatory issues/science policy; and integration of biomarkers into clinical design/qualification [4].

### Immunologic Monitoring: Standardization and Validation of Assays

Lisa H. Butterfield, PhD (University of Pittsburgh) chaired a session on standardization and validation on assays for immunological monitoring and delivered the first presentation in the session. In this update from the 2009 iSBTc Workshop, Butterfield summarized work completed by the iSBTc/SITC Biomarkers Taskforce, which included the recent preparation of the society's position paper *Recommendations from the iSBTc-SITC/FDA/NCI Workshop on Immunotherapy Biomarkers *[3]. Road blocks to developing immunotherapy biomarkers are the inherent variability of patients, variability of collection and processing of their blood and tissues, of selection and conduct of assays, and of the information reported on samples and assays reported in clinical trial and biomarker study manuscripts. The Taskforce recommendations include suggestions for ways to minimize variability by using standardized methods for blood and tissue processing and banking; standardized functional assays, thorough reporting of details and controls in publications, and banking of not only blood and serum but also patient DNA, tumor cells and tumor RNA (to determine patient genotypes and tumor gene expression profiles), and sufficient blood and serum for testing novel developing assays and hypothesis generation.

Paul V. Lehmann, MD, PhD (Cellular Technology Limited, Shaker Heights, OH), discussed the challenges of T cell monitoring: determining what parameters to measure, how to measure them, and most importantly, how to measure parameters precisely and reproducibly. He focused on the milestones that have lead to the successful standardization of enzyme-linked immunosorbent spot (ELISPOT) assays. These milestones included: 1) the development of protocols for the freezing of peripheral blood mononuclear cells (PBMCs) without functional loss; 2) the development of a library of reference PBMCs for assay comparisons, qualification/validation, and harmonization across institutions; 3) the development of serum free media for all steps of PBMC processing and testing; 4) the development of objective, automated analysis; 5) the development of ELISPOT assay qualification, validation, and high throughput testing; and 6) the demonstration that a unified platform suffices for obtaining highly reproducible ELISPOT data across technicians and institutions.

Representing the Association for Cancer Immunotherapy (CIMT), Cedrik M. Britten, MD (University Medical Center of the Johannes Gutenberg-University and BioNTech AG, Mainz, Germany) presented on harmonization of immunological monitoring across institutions. Britten reviewed the CIMT Immunoguiding Program (CIP), a proficiency panel program with 40 participating laboratories in 12 European countries. The aims of this program are to promote: 1) quality assurance by providing immediate feed-back about performance relative to the group (or to a dynamic reference value); 2) assay harmonization by using the collected data to systematically investigate the performance of subgroups and deduce harmonization guidelines; and 3) protocol optimization by using the collected data to systematically identify critical process steps. Britten presented CIP recommendations for harmonization of ELISPOT, which included: refraining from using allogeneic antigen presenting cells (APCs), using triplicate wells for each antigen, introducing a resting time of the PBMCs before they are added to the ELISPOT plate, adding an optimal cell number per well (≥ 4 × 10^5 ^lymphocytes per well), using serum-free test conditions, and using a scientifically sound method for response determination. Large-scale harmonization initiatives may lead to dynamic reference values to rank test performance, increased comparability of results generated across institutions, and improved assay performance in a group, thereby potentially accelerating clinical development of new cancer immunotherapies.

Britten also discussed the Minimal Information About T cell Assays (MIATA) initiative, which is part of a larger effort of "Minimal Information" projects for different types of data sets. The assay harmonization efforts conducted over the past five years have led to the identification of several critical experimental process steps. As a consequence, MIATA was launched as a community driven reporting framework for T cell experiments [5]. Published reports of T cell experiments, suggested Britten, should include sufficient information on all critical test variables and process steps, as agreed upon by a panel of participants, through a web-based iterative process with broad input from the immunotherapy field. Session 1 finished with a panel discussion with the audience, led by Butterfield, Lehmann, Britten, Sylvia Janetzk, MD (Zellnet Consulting, Inc., Fort Lee, NJ) and the CIC, and Michael Kalos, PhD (University of Pennsylvania).

### Correlation of Immunity to Clinical Response and Potency Assays

In a session focused on correlating immunity to clinical responses and potency assays, chaired by Mary L. Disis (University of Washington), Raj K. Puri, MD, PhD (Division of Cellular and Gene Therapies, Office of Cellular, Tissue and Gene Therapies, CBER, FDA) first discussed the FDA's considerations on potency and immune monitoring for cancer vaccines and cancer immunotherapy products. He discussed the importance of full product characterization, including development of potency assays according to FDA regulations, in successful product development. Puri discussed approaches for potency measurements, including 1) direct measurement of biological activity with *in vitro *or *in vivo *bioassays; 2) indirect measurement (i.e., surrogate assay) of biological activity using analytical, non-bioassays that are correlated to biological activity; and 3) the combination of multiple assays (a combination of biological or analytical assays where the combined results constitute an acceptable potency assay). Successful potency assays indicate biological activity(s) specific and relevant to the product and measure activity of all components deemed necessary for *in vivo *activity. Potency assays must provide a quantitative readout, indicate product stability, and meet predefined acceptance and/or rejection criteria. Results must be available in time for lot release. Importantly, fully-developed potency assays are required prior to the initiation of Phase 3 clinical trials so they may be validated during Phase 3 trials.

Puri summarized possible approaches to the successful development of potency assays, emphasizing the need to identify functional biomarkers (e.g., biomarkers that correlate with in vitro differentiation and/or detect functional cells in complex mixture). These may include the development of genomic or proteomic techniques to identify functional biomarkers, assessment of unique biochemical markers and secreted proteins, and/or flow cytometric assessment of cell phenotype for purity, which may link to identity and/or potency.

Immunological monitoring during development and evaluation of cancer immunotherapies can support proof of concept, advance understanding of immunological mechanisms (including T cell responses and modulation of regulatory cells), and provide information on mechanisms of action. Indeed, an immune response may correlate with clinical benefit, harm, or lack of either; thus immune monitoring may play a significant role in both early and late phases of immunotherapy product development. The FDA has drafted guidance documents for industry and for therapeutic cancer vaccines [6,7]. Additional references for the regulatory process for the Office of Cellular, Tissue, and Gene Therapies (OCTGT) for manufactures are available from the FDA [8].

Immunologic biomarkers as correlates of clinical response after cancer immunotherapy were presented by session chair, Mary L. Disis, MD. Citing recent data from clinical trials and population-based studies that have correlated biomarkers with clinical outcomes, Disis identified unifying themes around what constitutes an effective anti-tumor response, immunity types and the tumor microenvironment. For example, there is a strong correlation between gene expression in type I T cells (T_H1 _cells) and relapse in colorectal cancer [9] and the density of intratumoral T cells and overall survival in ovarian cancer [10]. Moreover, the composition of tumor-infiltrating T cells is associated with clinical outcomes; higher CD8^+^/CD4^+ ^T cell ratios and CD8^+^/T reg^+ ^ratios are independent predictors of survival in ovarian cancer [11].

Effective anti-tumor immunity also correlates with measurable changes in the tumor microenvironment following cancer immunotherapy. Modulation of self-regulation within the tumor is associated with response, as exemplified by the correlation between low T reg cell density within ER^+ ^breast cancer tumors [12]. Modulations of immune evasion within the tumor microenvironment are likewise linked to response, with high levels of PD-L1 expression correlating with lower density of CD8^+ ^T cells and survival in ovarian cancer [13]. Growth-factor mediated changes within the tumor microenvironments are also predictive of outcomes; lower TGFβ-1 levels within the tumor independently predicted longer disease free survival (DFS) among patients with breast cancer [14]. Functional persistence is also associated with an effective anti-tumor response, with higher density of CD45RO^+ ^memory T cells within the tumor independently predicting DFS among patients with colorectal cancer [15].

As a unifying theme surrounding immunological biomarkers of clinical response after cancer vaccine and T cell therapy, Disis emphasized that Type I immunity facilitates cross-priming and that autoimmunity is the ultimate endpoint of effective cross-priming. While current biomarker candidates generally focus only on the treatment-induced immune response, the impact of therapy on the tumor microenvironment may best predict maintenance of the induced immune response. Newer approaches that integrate measurement of effectors and environmental impact need to be fully assessed and larger studies are needed to demonstrate stronger associations between biomarkers and clinical response after cancer immunotherapy.

David Stroncek, MD (National Institutes of Health, Clinical Center) presented on measuring the potency of dendritic cell preparations using transcriptional analysis. Stroncek noted the importance of identifying biomarkers for new cellular therapies that can be used to assess: 1) consistency i.e., technical validation, including method validation (assays) and process validation (manufacturing); 2) biological variability, including inter-individual variability associated with genetic, epigenetic and clinical conditions, and intra-individual variability associated with changes in an individual over time or changes in health status. Potency biomarkers must discriminate between a biologically active and inactive product with minimal assay variability and accurately reflect manufacturing and individual variability. Stroncek et al are engaged in identifying biomarkers to assess mature dendritic cells (DCs). Standard phenotypic markers are useful for assessment of DC identity and purity, but not functional analysis. Stroncek reported on RNA microarray strategies for assessing patterns in DC gene expression that could be correlated with assay variability, manufacturing variability, and inter- or intra-donor variability. He provided examples of different levels of the expression of several immune response genes (e.g., CCL1, AIM2, and CD80) associated with these classes of variability. Stroncek's group is refining this strategy to systematically characterize cellular therapy potency biomarkers that reflect product consistency as well as individual and manufacturing variability. Dendritic cells are particularly challenging due to their environmental responsiveness, and thus, their phenotypic and functional changes during manufacture. Stroncek et al are using the concepts of this broad approach to design validation studies during clinical trials.

Sipuleucel-T immune parameters and correlation with overall survival was presented by Mark W. Frohlich, MD (Dendreon Corporation, Seattle, WA) based on recently reported results from the randomized Phase 3 IMPACT Trial (Immunotherapy Prostate AdenoCarcinoma Treatment) [16]. Immunological monitoring included assessment of product potency measures (i.e., CD54 upregulation as a marker of APC activation) and measures of cellular and humoral response. After the initial treatment with Sipuleucel-T, APC activation increased, indicated by CD54 upregulation, as did secretion of Type 1 cytokines. Proliferation and ELISPOT assays demonstrated specific T cell responses to the immunizing antigen after the initial dose. Sipuleucel-T was also shown to generate a persistent antigen-specific humoral response, which was characterized by antibody class switching from IgM to IgG (for anti-PA2024). In a combined analysis of Phase 3 Sipuleucel-T data, CD54^+ ^cell counts, number of total nucleated cells, and CD54 upregulation correlated significantly with overall survival, even after adjustment for baseline prognostic factors (PSA and LDH levels). The IMPACT study revealed a correlation between overall survival and measures of an antigen-specific antibody response, T cell proliferation, and ELISPOT.

The APC activation and cytokine profile associated with Sipuleucel-T is suggestive of an immunological prime-boost mechanism. The correlation between overall survival and the monitored immunological parameters suggests these measures may be useful biomarkers for assessing the clinical activity of this new cancer immunotherapy. Session 2 finished with a panel discussion led by Disis, Puri, Stroncek, Frohlich, Leif Håkansson, MD, PhD (Biotherapy Development Association) and Nicholas Restifo, MD (NCI Surgery Branch).

### Novel Methodologies for Assessing the Immune Landscape: Clinical Utility of Novel Technologies

The iSBTc/SITC Biomarkers Symposium included a session designed to address emerging methodologies that are proving useful in immune assessment for clinical immunotherapeutic approaches to cancer treatment chaired by Francesco Marincola (NIH) and Peter P. Lee (Stanford University). Thomas R. O'Brien, MD (National Cancer Institute, Division of Cancer Epidemiology and Genetics) presented on genetic variants in *IL28B *(IFN-λ) as major predictors of response to IFN-α therapy for chronic hepatitis virus C (HCV). Chronic HCV infection is the leading cause of liver cancer in the United States today. Standard treatment of chronic HCV infection involves pegylated IFN-alfa in combination with ribavirin, a regimen that generates a sustained virological response in about half of infected patients but which can have significant adverse effects. Use of appropriate markers and technologies to identify patients less likely to benefit from standard HCV treatment would be beneficial, as would more effective treatment approaches among these patients.

O'Brien reported on genome-wide association studies (GWAS) that have helped to link genetic variants in *IL28B *(which encodes IFN-λB) with the response to standard therapy. Analysis of global distribution of two *IL28B *alleles that differ by only a single nucleotide suggests that the higher frequency of the unfavorable allele within populations of African descent partially explains racial differences in response to standard treatment, pointing to a potential clinical role for IFN-λ in chronic HCV infection. While *IL28B *genotype may be helpful in indentifying patients who are not good candidates for therapy, personalized clinical decisions must consider other factors (e.g., viral load and hepatic fibrosis score) associated with a sustained virological response.

Samuel C. Silverstein, MD (Columbia University) presented data and mathematical models that indicate that a critical concentration of cytolytically active, tumor antigen-specific CD8^+ ^T cells is required to control growth of cognate antigen-expressing tumor cells. Silverstein described a clonogenic assay in which varying numbers of CD8^+ ^T cells from an OT-1 transgenic mouse whose T cell receptor specifically recognizes SIINFEKL peptide were mixed with B16 mouse melanoma cells (previously pulsed with SIINFEKL peptide) and co-incubated in a collagen/fibrin gel for 24, 48 and 72 hours. The gel was dissolved, the surviving cells plated, and the resulting colonies were counted to determine the number of surviving melanoma cells. In the absence of specific CD8^+ ^T cells, the melanoma cells demonstrate log-linear growth. With increasing numbers of co-incubated CD8^+ ^T cells, the melanoma cell growth rate is reduced, and at a critical CD8^+ ^T cell concentration, the cytolytic cells kill the tumor cells at the same rate as tumor cell growth. Silverstein reported on a mathematical model for determining killing efficiency in which the constant *k *was equal to the volume of antigen-expressing tumor cells cleared per cytolytically active, tumor antigen-specific CD8^+ ^T cell per minute. He presented killing efficiencies for *in vitro *(collagen-fibrin gels) and *in vivo *models (spleen cells of mice infused with LCMV-pulsed target cells) and demonstrated that *k *decreases 0.7 log_10 _for every log_10 _increase in CD8^+ ^T cell concentration and was dependent on the percent of *cytolytically active*, antigen-specific CD8^+ ^T cells present in the CD8^+ ^T cell milieu.

Jérôme Galon, PhD (INSERM, Integrative Cancer Immunology Laboratory, Cordeliers Research Center) presented on immune biomarkers, drawing from work that demonstrated that the immune contexture (nature, functional orientation, density and location of immune cells in colorectal cancer) had a prognostic value that was superior to that of the classic UICC-TNM classification system. He reviewed data that indicated that the presence of memory T cells within the tumor correlates with the absence of early-metastatic invasion and improved clinical outcome in colorectal carcinoma. He also discussed the prognostic value of tumor invasion vs. immune reaction, demonstrating an inverse relationship between intratumoral density of CD8^+ ^T cells and the T stage of the in colorectal carcinoma tumor at the time of surgery. Moreover, data he summarized indicated that most patients with a strong and coordinated cytotoxic response presented with early-stage colorectal carcinoma, whereas patients with a weak cytotoxic response progressed to late-stage disease. Additionally, the density of CD8^+ ^T cells at the center of the tumor also correlated inversely with tumor T stage and relapse.

Peter P. Lee, MD (Stanford University) presented information on the assessment of immune changes in tumor-draining lymph nodes (TDLNs) as novel biomarkers using an integrated image analysis approach. Using 5-color immunohistochemical staining, automated high-resolution (whole section) imaging, and customized image analysis software, Lee's group have been able to create composite images that map each cell type within sections of TDLNs. The number, proportion, and spatial characteristics (i.e., spatial relationships between immune and tumor cells) were compared to five year clinical outcome data. Lee reported changes in immune cells in TDLNs, both in number and spatial relationship, and that some of these changes appear to predict clinical outcome. He noted that quantitative, spatial analysis tools for histology have been developed for high throughput analysis, thus image analysis of immune cells in TDLNs may serve as a novel biomarker for cancer. Initial analysis of TDLNs from patients with breast cancer suggests that this approach may also have broader utility in other cancers. Session 3 finished with a panel discussion led by Marincola, Lee, O'Brien, Silverstein, and Galon.

### Recommendations on Incorporation of Biomarkers into the Clinical Arena

The final session geared toward providing insight into the incorporation of biomarkers into clinical applications was chaired by John M. Kirkwood, MD (University of Pittsburgh). First, Diane Longo, PhD (Nodality, Inc., Foster City, CA) presented on single cell network profiling (SCNP) technology and applications in immunological monitoring. This technology, based on multiparameter flow cytometry, provides measurement of both extracellular surface markers and intracellular signalling within single cells. This approach can be used to distinguish basal, unevoked subsets of cells from evoked cells after clinically-relevant stimulation, making it useful for immunological monitoring. SCNP technology may help in disease characterization by mapping deregulated pathways. In pre-clinical drug profiling efforts, SCNP may be useful in characterizing drug potency, target selectivity, and off-target activity, and resistance. Additionally, SCNP may assist in patient stratification and individual patient drug profiling. Thus, interrogation of cell signaling with SCNP allows a direct means to classify disease activity and response to treatment. The relationships of signaling events to each other can be used to infer a structure to the immune system, providing useful immunological information during development and clinical testing of immunotherapies.

Daniel Normolle, PhD (University of Pittsburgh Cancer Institute) presented on biostatistical considerations for biologics and biomarkers in oncology, summarizing the limitations of the 3 + 3 design of early phase clinical studies and outlining alternative designs that include immunotherapy biomarkers. Among the limitations of the 3 + 3 trial design, often used in early clinical trials of biological therapies of cancers, is that this study design is intended for treatments in which toxicities increase with dose. A large proportion of participants are treated with sub-therapeutic doses. This study design can results in a slow dose escalation even when no dose limiting toxicities are observed and there is no quantitative mechanism to employ prior understanding of toxicities in the design. While the 3 + 3 design can eliminate harmful doses from further testing, it is underpowered for selecting among the remaining doses. Thus, while this design can eliminate extremely toxic doses, it does not choose between doses that are not extremely toxic and is less suited for evaluation of biological therapies that have low toxicities or toxicities that do not increase with dosing.

In the context of non-cytotoxic biological therapies, monitoring toxicity is distinct from escalating dose based on toxicity. In the 3 + 3 design, if toxicity is low with a given dose, the dose is automatically moved to the next highest dose, which may not be the best therapeutic dose. Moreover, if an added component reduces toxicity, escalating dose on toxicity may again fail to choose the most useful dose. Importantly, cohorts of 3 and 6 patients are often too small to provide meaningful statistical information to guide dosing decisions.

Normolle outlined an alternate, adaptive design to escalating dose based on toxicities which incorporated the assessment of biomarkers. The alternate early trial design should be constructed to provide information to prove the principle and identify sources of variability in biomarker assessment. It should estimate the biologically effective doses and eliminate ineffective doses as well as provide information on the relationships between biomarkers at biologically effective doses. An adaptive trial design of immunotherapies should establish immunological activity at the highest dose and determine if lower doses are as effective as the highest dose, while avoiding ineffective doses. Toxicity must be monitored and a global stopping rule for toxicity should be in place. In randomized trials, participants should be allocated equally to the dosing arms of the study. The studies can be designed as simple randomized trials, two- or three-staged randomized trials or as trials of combination therapy to reduce toxicity. It is critical that the trial be statistically powered to achieve the primary objective of the study.

Holden T. Maecker, PhD (Stanford University) discussed prospects for new clinical flow cytometry assays. While clinical tests for cellular immunity are largely lacking, flow cytometry represents a powerful technology for dissecting cellular immune responses. In assessing immune responses it is useful to determine the number of functional and non-functional T cells specific to a particular antigen. Qualitative information on T cells to a specific antigen is also invaluable. Such qualitative information may include the breadth of epitopes recognized, the types of cytokines produced, degranulation or lytic capacity, and phenotypic markers on the T cells (e.g., memory/effector markers, markers of exhaustion [PD-1], perforin, granzymes). Flow cytometry can provide much of this information because it can used to measure multiple markers on individual cells, detect rare cell populations, and can measure both cellular phenotypes and functions.

Intracellular cytokine staining (ICS) has been simplified and standardized for flow cytometry using plates with lyophilized antigen. This approach has been useful in dissecting the cytokine profile of various T cell subsets in response to HIV and cytomegalovirus. Phospho-Flow assays are useful for the assessment of intracellular signaling as they can measure phosphorylation events in very short-term stimulated whole blood, PBMC, and other cells. These assays can measure multiple cell-surface and intracellular markers in combination, using multiparameter flow cytometry and detect signaling through T cell receptors, surface Ig, cytokines and other molecules. Phospho-Flow assays may be used to detect signaling defects in aging or immune-mediated diseases. Flow cytometry can provide useful information on early and late cellular immune responses and may have clinical utility in the assessment of cellular changes in response to various disease and treatment. Simplification and standardization of methodology will be necessary for clinically useable tests [17].

In the final presentation, Howard L. Kaufman, MD (Rush University) discussed predictive biomarkers for tumor immunotherapy and whether the community is ready for clinical implementation. Kaufman outlined requirements for an ideal biomarker--that it correlate with disease progression or treatment response, be easily collected and accurately measured, that it be validated, and that it be cost-effective. Biomarkers may be useful for monitoring adverse events, identifying potential targets for drug discovery, and informing decisions about clinical trials, including selection of patients, endpoints and dosing. In immunotherapy studies, biomarkers have included soluble factors (e.g., serum proteins, circulating DNA, circulating tumor cells), tumor factors (e.g., receptor expression, cellular infiltrates), patients factors (indicators of humoral and cellular immune responses, immune system polymorphisms) and mathematical predictions. Cancer immunotherapy trials have included CD4^+^, CD8^+ ^T cell responses, Treg responses and antibody titre as predictors for clinical response. The utility of these biomarkers has been limited by the small size of most of these trials, limited clinical response and by the fact that biomarker analysis is often retrospective and unplanned for in the trial design.

A number of biomarkers have been evaluated in IL-2 immunotherapy in renal cell carcinoma, including pre-treatment leukocyte and neutrophil levels, Ki-67 expression, CAIX levels, VEGF levels, clonal T cell expansion, and levels of CD4^+^CD25^hi ^Treg cells. Kaufman et al have employed a computational model that includes density and distribution of the IL-2 receptor in conjunction with delivered IL-2 dose to predict the clinical response to IL-2 immunotherapy for renal cell carcinoma. These computational biomarkers and other potential soluble and cellular biomarkers warrant incorporation into prospective clinical trials of cancer immunotherapies and further validation in larger trials. The session finished with a panel discussion led by Kirkwood, Longo, Normolle, Maecker and Kaufman.

In summary, the Symposium speakers presented promising new data on emerging immune biomarkers in cancer. Several themes recurred through many of the presentations: first, standardization and harmonization efforts have identified critical parameters in patient sample handling and assay conduct and reporting; second, we are observing clinical and subclinical autoimmunity in treated patients as well as extensive responses to self tumor antigens, which may indicate the critical role for *in vivo *cross-presentation; third, there were examples of large scale trials in which biomarkers were examined not only in blood, but also in tumor and lymph nodes, which were highly significantly correlated to clinical outcome; and fourth, that the labs, taskforces, and societies represented were all participating in overlapping collaborations, indicating the success of working together. Intensive interaction between academia, industry and government--as represented in this iSBTc/SITC symposium--is necessary to promote the development of predictive biomarkers for improved cancer outcomes through immunotherapy.

## Competing interests

MLD discloses the following relationships: Glaxo, Grant Funding, Principal Investigator; Hemispherex, Grant Funding, Principal Investigator; and VentiRx, Consulting Fee, Consultant. LHB, SNK, JB and FM declare that they have no competing interests.

## Authors' contributions

LB, MD, SK and FM: planned, organized, and chaired the Symposium; JB: drafted the manuscript; LB: critically reviewed and edited the manuscript and prepared the bibliography; All authors read and approved the final manuscript.

## Appendix

Organizations represented at the 2010 SITC Collaboration Summit included Biotherapy Development Association (BDA), Canadian Cancer Immunotherapy Consortium (CCIC), Association for Cancer Immunotherapy (CIMT), Cancer Immunotherapy Consortium, a program of the Cancer Research Institute (CRI-CIC), Chinese Society of Clinical Oncology (CSCO), European Society for Cancer Immunology and Immunotherapy (ESCII), Japanese Society of Clinical Immunology (JSCI), Nordic Center for Development of Antitumour Vaccine Concept (NCV-Network), and the Italian Network for Tumor Biotherapy (NIBIT).
